# Supplementary material for: Three-stage ultrafast demagnetization dynamics in a monolayer ferromagnet
Source: Nat Commun. 2024 Mar 30;15:2804. doi: 10.1038/s41467-024-47128-4 (PMC10981666; doi:10.1038/s41467-024-47128-4)
Supplement: Supplementary file 1 — Supplementary Information [file 41467_2024_47128_MOESM1_ESM.pdf]

Supplementary Information for

Three-stage ultrafast demagnetization dynamics in a monolayer ferromagnet

Na Wu,[1,2,*] Shengjie Zhang,[1,2,*] Daqiang Chen,[1,2] Yaxian Wang,[1,†] and Sheng Meng[1,2,3,‡]

[1]*Beijing National Laboratory for Condensed Matter Physics and Institute of Physics, Chinese Academy of Sciences, Beijing 100190, China*

[2]*School of Physical Sciences, University of Chinese Academy of Sciences, Beijing 100190, China*

[3]*Songshan Lake Materials Laboratory, Dongguan, Guangdong 523808, China*

(Dated: March 11, 2024)

[*] These authors contributed equally to this work.

[†] Email address: yaxianw@iphy.ac.cn;

[‡] Email address: smeng@iphy.ac.cn;

TABLE OF CONTENTS

Section S.1 Time-dependent density functional theory

Figure S1. Waveform of the applied electric field.

Section S.2 TDDFT in the plane wave basis and the evolution operator

Section S.3 The spin-lattice coupling in bulk FGT

Figure S2. The spin-phonon interaction in bulk FGT.

Section S.4 The FFT analysis for the magnetization dynamics

Figure S3. The FFT spectrum of the magnetization oscillation during 200–800 fs.

Section S.5 A complete presentation of the in-plane phonon dynamics

Figure S4. The time-dependent in-plane phonon dynamics.

Section S.6 The magnetization dynamics to 1250 fs

Figure S5. The time-dependent magnetization dynamics for the time range 0–1250 fs.

Section S.7 Role of electron excitation in the spin-phonon coupling

Figure S6. The role of electron excitation.

Section S.8 Magnetization dynamics contributed from different atoms

Figure S7. The atom-resolved magnetization dynamics of FGT.

Section S.9 Nonlinear phononics under different pump fluences

Figure S8. The excited A_{1g} and E_{2u} phonon amplitudes with increasing laser fluence.

Figure S9. The different behaviors of in-plane phonons under weak and intense excitation.

S.1. TIME-DEPENDENT DENSITY FUNCTIONAL THEORY

The time evolution of electron wave functions is governed by the time-dependent Kohn-Sham (TDKS) equation [1]:

$$i\frac{\partial}{\partial t}\psi_{\gamma,\mathbf{k}}(\mathbf{r},t) = \left[\frac{1}{2m}(\mathbf{p}-\frac{e}{c}\mathbf{A})^2 + V(\mathbf{r},t)\right]\psi_{\gamma,\mathbf{k}}(\mathbf{r},t), \tag{S1}$$

where velocity gauge is used and the external field appears in the kinetic term in the form of vector potential $\mathbf{A}(t)$.

The propagation of TDKS orbitals is implemented on the adiabatic basis $\phi_{n,\mathbf{k}}(\mathbf{r},t)$

$$\left|\psi_{\gamma,\mathbf{k}}(\mathbf{r},t)\right\rangle = \sum_n c_{n\gamma,\mathbf{k}}(t)\left|\phi_{n,\mathbf{k}}(\mathbf{r},t)\right\rangle, \tag{S2}$$

where γ and n denote the TDKS band index and the basis index, respectively. $\mathbf{k}$ refers to the reciprocal momentum index and $c_{\gamma n,\mathbf{k}}(t)$ the time dependent coefficients. The adiabatic basis is calculated on the fly at each ionic step by diagonalizing the Hamiltonian:

$$H_{\mathbf{k}}(\mathbf{r},t)\left|\phi_{n,\mathbf{k}}(\mathbf{r},t)\right\rangle = \varepsilon_{n,\mathbf{k}}(t)\left|\phi_{n,\mathbf{k}}(\mathbf{r},t)\right\rangle, \tag{S3}$$

where $\varepsilon_{n,\mathbf{k}}(t)$ is the eigenvalue. Thus, the charge density can be calculated with $c_{\gamma n,\mathbf{k}}(t)$ and $\phi_{n,\mathbf{k}}(\mathbf{r},t)$:

$$\begin{aligned}\rho(\mathbf{r},t) &= \sum_{\mathbf{k}}\sum_{\gamma}^{occ} f_\gamma \psi^*_{\gamma,\mathbf{k}}(\mathbf{r},t)\psi_{\gamma,\mathbf{k}}(\mathbf{r},t)\\ &= \sum_{\mathbf{k}}\sum_{nm}\sum_{\gamma}^{occ} c^*_{n\gamma,\mathbf{k}}(t)c_{m\gamma,\mathbf{k}}(t)\phi^*_{n,\mathbf{k}}(\mathbf{r},t)\phi_{m,\mathbf{k}}(\mathbf{r},t),\end{aligned} \tag{S4}$$

where f_γ represents the occupation number of time-dependent Kohn-Sham wavefunctions.

For ions that are much heavier than electrons, their motions are treated classically on an averaged potential energy surface determined by the electronic distribution according to the Ehrenfest theorem. The nuclear positions are updated following the Hellmann-Feynman theorem [2]:

$$M_\alpha\frac{d^2\mathbf{R}_\alpha}{dt^2} = -\sum_\gamma f_\gamma\langle\psi_\gamma|\nabla_{\mathbf{R}_\alpha}(\frac{1}{2m}(\mathbf{p}-\frac{e}{c}\mathbf{A})^2 + V(\mathbf{R},\mathbf{r},t))|\psi_\gamma\rangle \tag{S5}$$

where M_α and $\mathbf{R}_\alpha$ are the mass and position of the αth ion. Eq. (S1) and Eq. (S5) represent the coupled electron-ion motion.

The Gaussian-envelop laser pulse we applied has the following waveform.

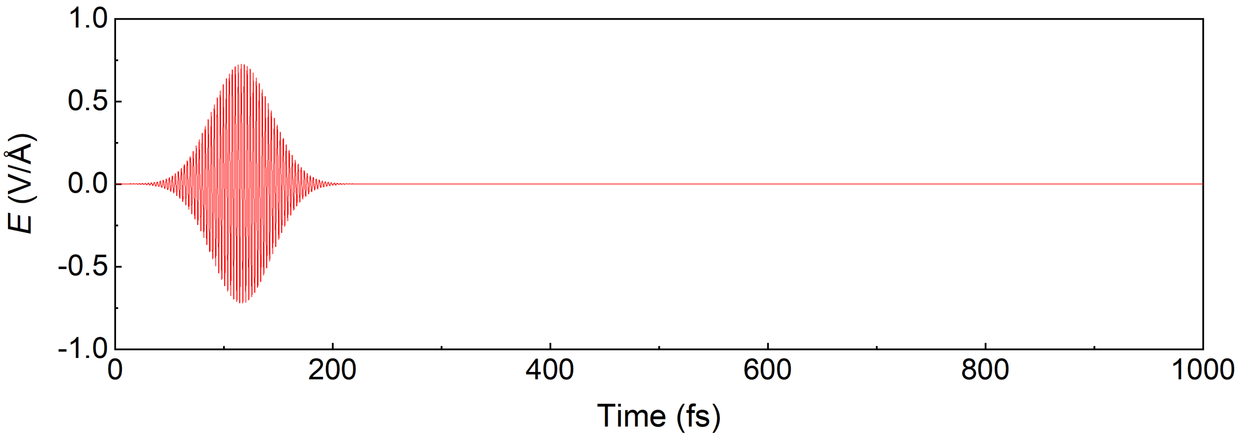

FIG. S1. Waveform of the applied electric field linearly polarized along the c-axis of the crystal cell.

S.2. TDDFT IN THE PLANE WAVE BASIS AND THE EVOLUTION OPERATOR

In the representation of plane wave (PW) basis sets $\{\mathbf{G}\}$, the time-dependent Kohn-Sham states $\psi_{\gamma,\mathbf{k}}(\mathbf{G},t)$ at each $\mathbf{k}$ point can be expanded in the adiabatic basis sets $\{\phi_{n,\mathbf{k}}(\mathbf{G},t_1)\}$, which are the eigenstates of Hamiltonian $H_{\mathbf{k}}(\mathbf{G},t_1)$ [3, 4]:

$$\left|\psi_{\gamma,\mathbf{k}}(\mathbf{G},t)\right\rangle = \sum_{\gamma} c_{n\gamma,\mathbf{k}}(t)\left|\phi_{n,\mathbf{k}}(\mathbf{G},t_1)\right\rangle, \tag{S6}$$

where the coefficient $c_{n\gamma,\mathbf{k}}(t) = \left\langle \phi_{n,\mathbf{k}}(\mathbf{G},t_1) \mid \psi_{\gamma,\mathbf{k}}(\mathbf{G},t)\right\rangle$. Writing $c_{n\gamma,\mathbf{k}}(t)$ in the form of the coefficient matrix $C_{\mathbf{k}}(t)$, the time-dependent Kohn-Sham (TDKS) equation in the adiabatic basis $\{\phi_{n,\mathbf{k}}(\mathbf{G},t_1)\}$ is

$$H_{\mathbf{k}}(t)C_{\mathbf{k}}(t) = i\hbar\frac{\partial C_{\mathbf{k}}(t)}{\partial t}. \tag{S7}$$

At an infinitesimal time interval ($\Delta t = t_2 - t_1$), the Hamiltonian in the adiabatic basis can be regarded as varying linearly with time

$$H_{\mathbf{k}}(t) = H_{\mathbf{k}}(t_1) + \frac{t-t_1}{t_2-t_1}\left[H_{\mathbf{k}}(t_2) - H_{\mathbf{k}}(t_1)\right]. \tag{S8}$$

Using the evolution operator $U_{\mathbf{k}}(t_2,t_1)$, the Eq. S7 can be written as

$$C_{\mathbf{k}}(t_2) = U_{\mathbf{k}}(t_2,t_1)C_{\mathbf{k}}(t_1) \tag{S9}$$

According to Crank-Nicholson algorithm, $U_{\mathbf{k}}(t_s+dt,t_s)$ can be expanded as

$$U_{\mathbf{k}}(t_s+dt,t_s) = \exp(-i\hbar H_{\mathbf{k}}(t')dt/2) \approx \frac{1-i\hbar H_{\mathbf{k}}(t')dt/2}{1+i\hbar H_{\mathbf{k}}(t')dt/2}, \tag{S10}$$

with $t' = t_s + dt/2$. Under the condition of sufficiently small step ($dtH \ll 1$), we interpolate N_t (in our case $N_t = 1000$) copies during the time range $\Delta t = t_2 - t_1$, *i.e.* $t_s = t_1 + sdt$ and $dt = \Delta t/N_t$. Now that $U_{\mathbf{k}}(t_2, t_1)$ can be expressed as:

$$\begin{aligned} U_{\mathbf{k}}\left(t_2, t_1\right) &= \prod_{s=0}^{N_t-1} U_{\mathbf{k}}\left(t_s + dt, t_s\right) \\ &\approx \prod_{s=0}^{N_t-1} \frac{1 - i\hbar H_{\mathbf{k}}\left(t_s + dt/2\right) dt/2}{1 + i\hbar H_{\mathbf{k}}\left(t_s + dt/2\right) dt/2}. \end{aligned} \tag{S11}$$

From Eq. S11, the time step of the ionic system is Δt while the time step of electronic systems is $dt = \Delta t/N_t$. Therefore in our calculations, the timesteps for the electronic and ionic systems differ by three orders of magnitude.

S.3. THE SPIN-LATTICE COUPLING IN BULK FGT

To gain a better understanding of the strength of spin-lattice coupling in the layered structure, we perform static calculations to obtain the variation of the magnetic moment in the z-direction (ΔM_z) in monolayer and bulk FGT for the phonon modes of our interest. The $A_{1\mathbf{g}}$ phonon modes in bulk FGT are demonstrated in Fig. S2, with the two layers having either in-phase or out-of-phase atomic motions. As shown in Fig. S2, the variation of magnetic moment upon the same phonon displacement of the in-phase $A_{1\mathrm{g}}$-1 phonon mode of bulk FGT is very similar to that observed in the monolayer (Fig. 3 in the main text). However, the spin-phonon coupling strength for the in-phase $A_{1\mathrm{g}}$-2 mode in bulk FGT is roughly one-fifth of that in the monolayer. This indicates that the interlayer interaction might change the electron screening in bulk metallic magnetic materials, which suppresses the spin-phonon coupling compared to that in the monolayer. This aligns with previous work where strong spin-phonon coupling is mostly observed in fewer-layer samples [5]. As for the out-of-phase $A_{1\mathrm{g}}$ phonons, the variation of magnetization (ΔM_z) on phonon displacement shows a quadratic dependence in bulk FGT, and the amplitude is at least one order of magnitude smaller, due to the cancellation effect between adjacent layers. Therefore, we believe the monolayer is an ideal platform to study the ultrafast spin dynamics and can reveal the fundamental physics involved, yet a full-scale TDDFT simulation on bulk and few-layer FGT is highly desirable that we leave for future investigations.

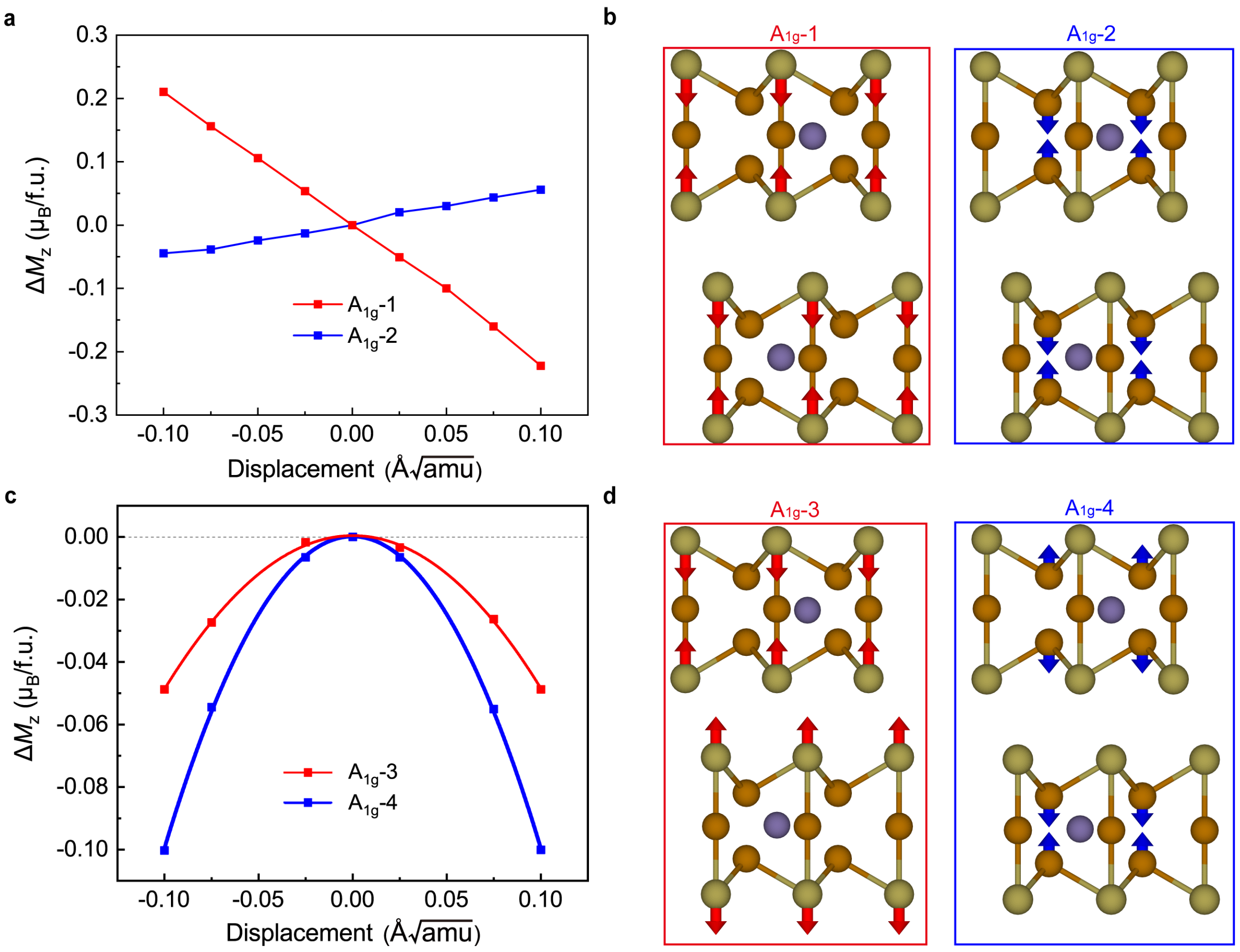

FIG. S2. **The spin-phonon interaction in bulk FGT.** Variation of M_z for the (a) in-phase and (c) out-of-phase A_{1g} phonon modes in bulk FGT, whose atomic displacements are shown in (b) A_{1g}-1 and A_{1g}-2; (d) A_{1g}-3 and A_{1g}-4, correspondingly.

S.4. THE FFT ANALYSIS FOR THE MAGNETIZATION DYNAMICS

As Fig. S3 shows, the FFT spectrum of the magnetization dynamics after 200 fs has been examined, in which there are two dominant peaks with frequency approximately $\omega_{\text{peak1}} = 4.72$ THz and $\omega_{\text{peak2}} = 8.89$ THz. The two frequency peaks correspond to the occurrence of the two A_{1g} phonon modes, which highlights the dominant role of A_{1g} phonon modes in the dynamics of the second-stage demagnetization. Besides, the two peak frequencies are slightly increased compared with the phonon eigenfrequency in the ground state (4.58 THz and 8.87 THz). These results are consistent with the phonon hardening effect in Fig. 5 in the main text.

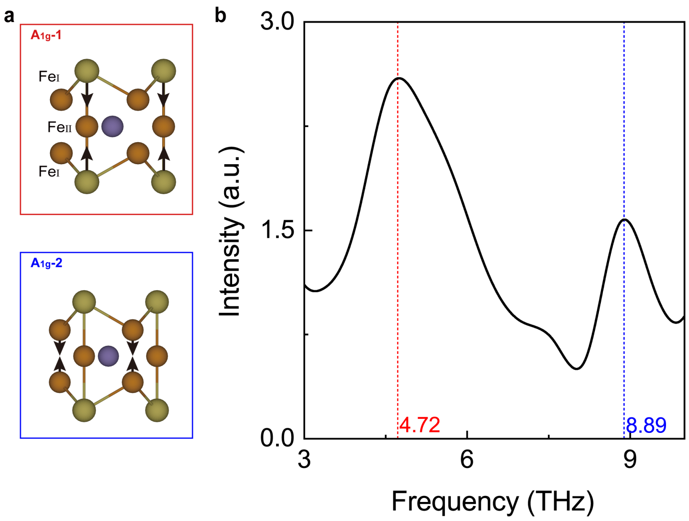

FIG. S3. **The FFT spectrum of the magnetization oscillation during 200–800 fs.** Two dominant FFT peaks of ω_{peak1} = 4.72 THz and ω_{peak2} = 8.89 THz are corresponding to the two A_{1g} phonon modes.

S.5. A COMPLETE PRESENTATION OF THE IN-PLANE PHONON DYNAMICS

To explicitly examine the in-plane lattice vibration, we show in Fig. S4 the time-dependent projected intensities for all phonon modes. More specifically, the in-plane Raman-active A_{2g} phonon modes (denoted by yellow and brown lines) are excited upon imposing the laser pulse. During the after-pulse dynamics, two sets of doubly-degenerat infrared active phonons (in green and purple lines) are activated and their amplitudes increase over time, especially for the modes in purple after 800 fs. This is likely arising from phonon coupling with the initially activated Raman-active modes with large amplitudes. Therefore, there exist the in-plane phonons throughout the process but the chiral ones which affect the magnetization dynamics start to play a dominant role in the demagnetization dynamics after 800 fs.

S.6. THE MAGNETIZATION DYNAMICS TO 1250 FS

Due to the substantial computational cost, we have carried out simulations of the temporal magnetization dynamics at a longer timescale to 1250 fs, shown in Fig. S5. We do observe a recovery of the z-component of magnetic moment M_z after 1000 fs. This is mostly likely due to the thermalization of lattice vibrations after 1 ps, from which the chiral phonon, or the circular motion

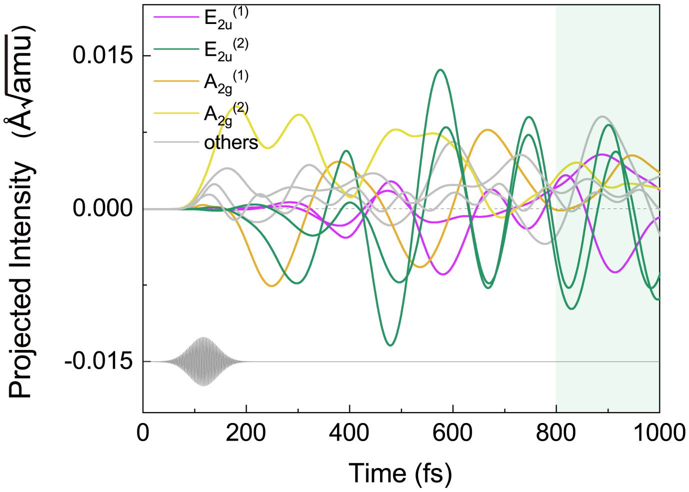

FIG. S4. **The time-dependent in-plane phonon dynamics.** The yellow and brown lines denote the Raman-active in-plane A_{2g} phonon modes; the green and purple lines denote two sets of doubly-degenerate infrared in-plane E_{2u} phonon modes.

of the Fe atoms becomes incoherent. This is an indication that the chiral phonons driven from nonlinear phononics might not maintain a strong coherence and the phase is strongly coupled with other phonon modes. If one can selectively drive the chiral phonons as proposed in for example Ref. [6], the spin precession could be better manipulated.

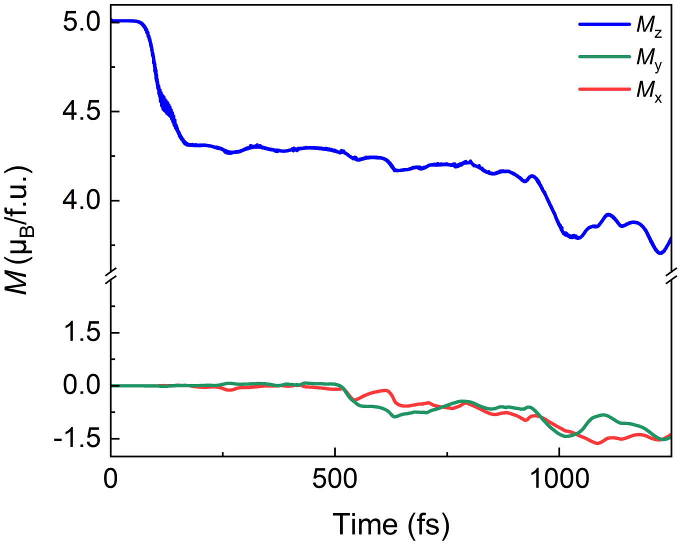

FIG. S5. **The time-dependent magnetization dynamics for the time range 0-1250 fs.** The red, green, and blue lines represent M_x, M_y, and M_z, respectively.

S.7. ROLE OF ELECTRON EXCITATION IN THE SPIN-PHONON COUPLING

To understand the suppressed spin-phonon coupling in the excited states, we make a direct comparison by tracking the magnetization dynamics when electron excitation is absent. Specifically, we take the transient lattice structure for each time step and calculate the magnetization, but taking the electron occupation numbers as in their *ground state*. As can be seen in Fig. S6, the magnetization did not show an obvious demagnetization but rather a fluctuation near the initial M_z value. Therefore, we believe the high electron temperature, or more specifically, the nonequilibrium electron distribution will affect the spin-lattice coupling.

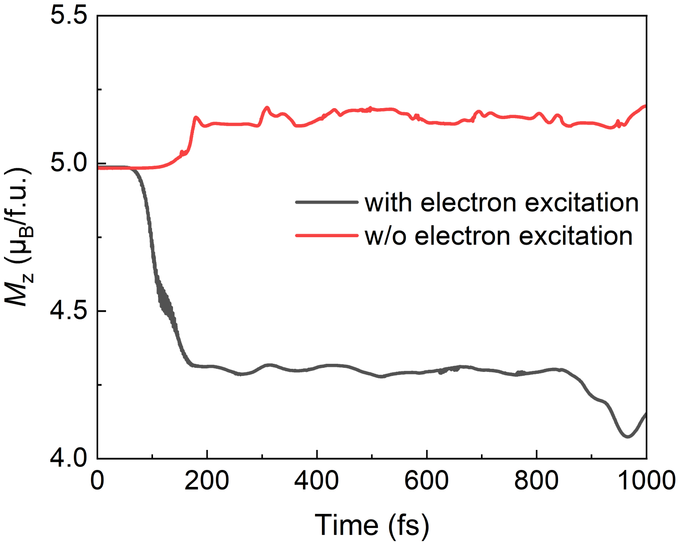

FIG. S6. **The role of electron excitation.** Light-driven magnetization dynamics with electron excitation (in black lines) and w/o electron excitation (in red lines).

S.8. MAGNETIZATION DYNAMICS CONTRIBUTED FROM DIFFERENT ATOMS

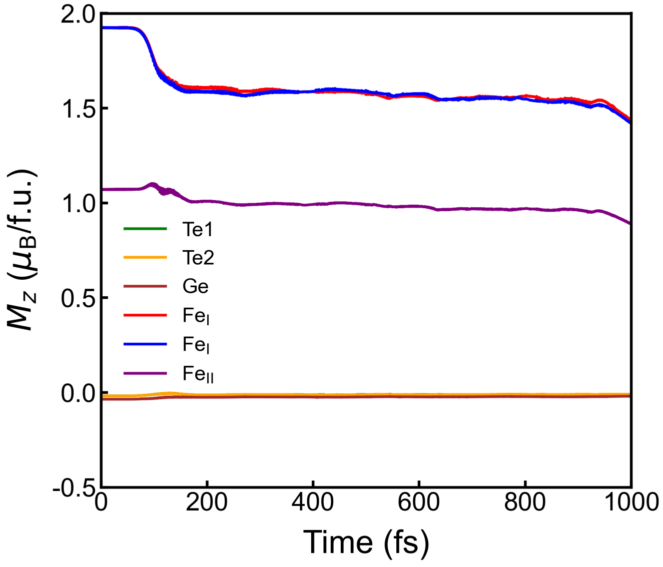

FIG. S7. **The atom-resolved magnetization dynamics of FGT.** The temporal evolution of the magnetic moment of each atom in the FGT primitive cell.

S.9. NONLINEAR PHONONICS UNDER DIFFERENT PUMP FLUENCES

In this section, we discuss in more detail the phonon and spin dynamics in the weak and intense excitation regimes. In the main text, we demonstrate distinctive magnetization dynamics under *weak* and *intense* excitations. Here, we show a quantitative evaluation on the dependence of A_{1g} amplitude on the pump fluence, where we see a quadratic increase in the phonon amplitude within the low-intensity regime shown in Fig. S8. In particular, it is worth noting that the distinction between the *weak* and *intense* excitation could also be seen from the amplitude of the in-plane E_{2u} phonons induced by the phonon-phonon interactions. Only when the pump fluence is beyond 1.93 mJ/cm^2 can the E_{2u} phonons reach a substantial amplitude to drive the chiral phonon and spin precession (labeled by the black and purple dotted lines in Fig. S8 to guide the eye).

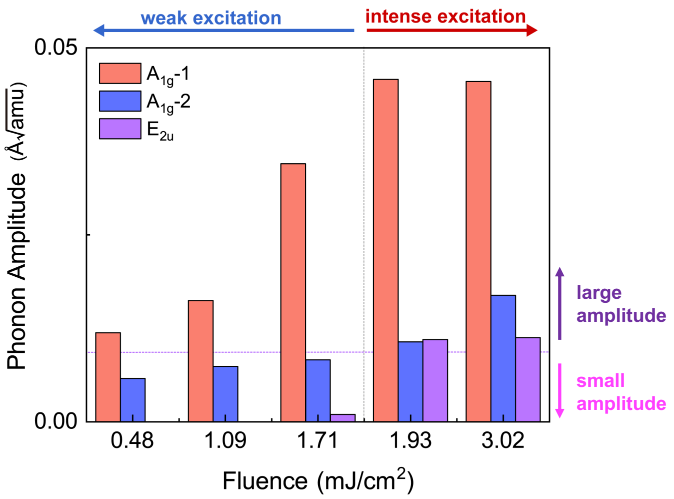

FIG. S8. The excited A_{1g} phonon amplitudes with increasing laser fluence, with a quadratic dependence at the low intensity limit. The purple blocks show the amplitude of the infrared-active in-plane E_{2u} phonon modes excited from nonlinear phononics, showing a *threshold* behavior. The black dotted vertical line indicates the threshold for the pump fluence, while the purple dotted horizontal line schematically shows the threshold for the E_{2u} phonon amplitude to induce chiral phonons and spin precession to guide the eye.

To better visualize such an effect, we shown the projected phonon intensity under three pump fluences in Fig. S9. As shown in the upper and middle panels of Fig. S9(a) and (b), under the fluence of 1.71 mJ/cm^2, the optically excited A_{1g} modes have a amplitude only a fraction of those under 1.93 mJ/cm^2, which leads to a rather small infrared-active in-plane A_{2g} modes, and as a result an absence of E_{2u} modes. Therefore, the increase in pump fluence determines the lattice

vibrations which are essential for the chiral phonon generation and the spin precession.

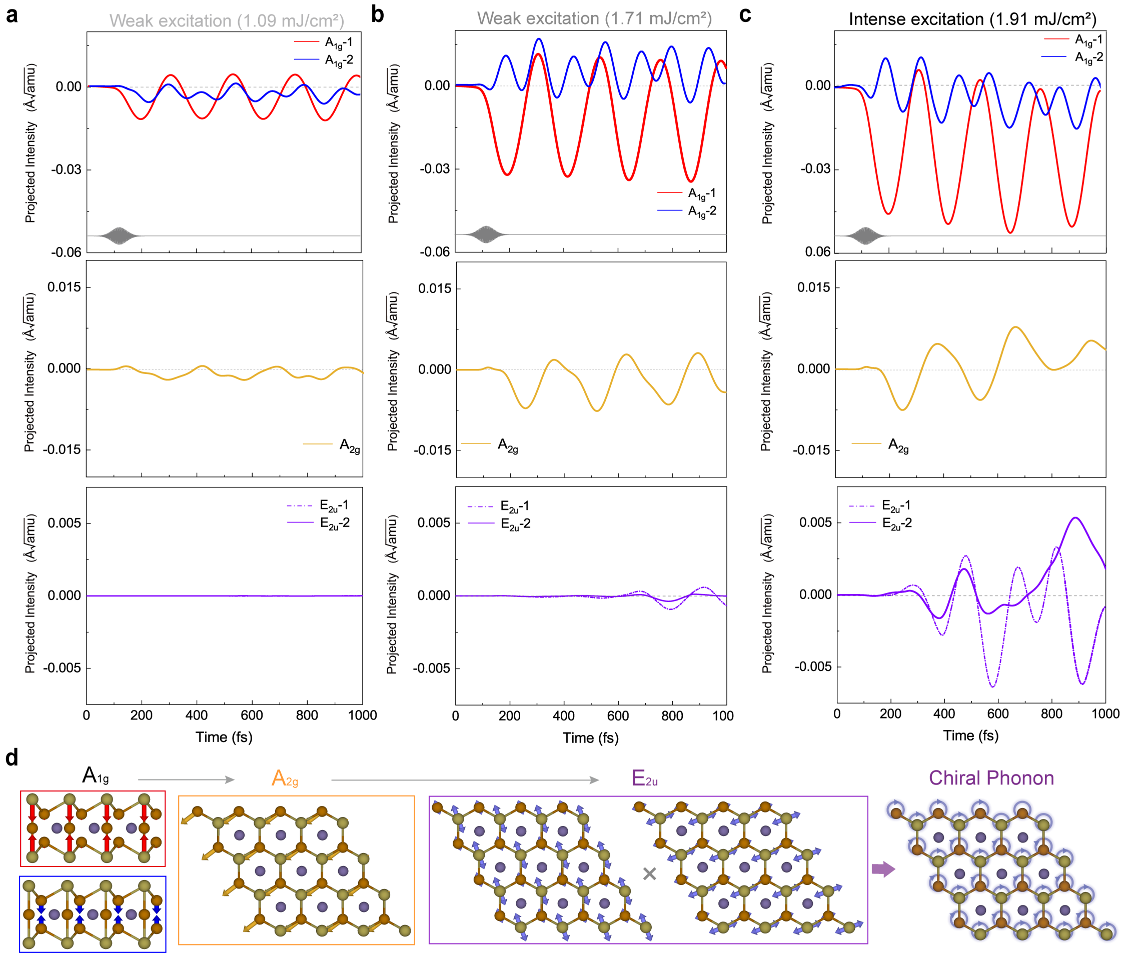

FIG. S9. **The different behaviors of in-plane phonons under weak and intense excitation.** The time-dependent phonon dynamics under laser fluence of (a) 1.09 mJ/cm^2, (b) 1.71 mJ/cm^2, and (c) 1.93 mJ/cm^2. The three rows, from top to bottom, correspond to the projected intensity of out-of-plane A_{1g}, in-plane A_{2g}, and in-plane E_{2u} phonon modes, respectively. The Ramman-active A_{2g} are coherently driven with frequencies of 106.72 cm^{-1}, shown by the yellow curves. After 300 fs, doubly degenerate infrared-active phonon modes E_{2u}, with frequencies of 76.18 cm^{-1} are driven due to the phonon-phonon interactions. (d) The eigenmodes of these large-amplitude phonons are visualized from the perspective along the positive direction of *a*-axis. The superposition of the doubly-degenerate E_{2u} modes can result in circular atomic motion, *i.e.* activating the chiral phonons. Under weak excitation, the E_{2u} modes have negligible amplitude thus leading to the absence of chiral phonons and spin precession.

REFERENCES

[1] E. Runge and E. K. U. Gross, Phys. Rev. Lett. **52**, 997 (1984).

[2] J. C. Tully, Faraday Discuss. **110**, 407 (1998).

[3] C. Lian, S.-J. Zhang, S.-Q. Hu, M.-X. Guan, and S. Meng, Nature Communications **11**, 43 (2020).

[4] P. You, D. Chen, C. Lian, C. Zhang, and S. Meng, Wiley Interdisciplinary Reviews: Computational Molecular Science **11**, e1492 (2021).

[5] X. Kong, T. Berlijn, and L. Liang, Advanced Electronic Materials **7** (2021), 10.1002/aelm.202001159.

[6] T. F. Nova, A. Cartella, A. Cantaluppi, M. Först, D. Bossini, R. V. Mikhaylovskiy, A. V. Kimel, R. Merlin, and A. Cavalleri, Nature Physics **13**, 132 (2017).
